# Effects of cell size and bicarbonate on single photon response variability in retinal rods

**DOI:** 10.3389/fnmol.2022.1050545

**Published:** 2022-12-14

**Authors:** Polina Geva, Giovanni Caruso, Colin Klaus, Heidi E. Hamm, Vsevolod V. Gurevich, Emmanuele DiBenedetto, Clint L. Makino

**Affiliations:** ^1^Department of Physiology and Biophysics, Boston University Chobanian & Avedisian School of Medicine, Boston, MA, United States; ^2^Italian National Research Council, Istituto di Scienze del Patrimonio Culturale, Roma, Italy; ^3^Mathematical Biosciences Institute, Ohio State University, Columbus, OH, United States; ^4^College of Public Health, Division of Biostatistics, Ohio State University, Columbus, OH, United States; ^5^Department of Pharmacology, Vanderbilt University, Nashville, TN, United States; ^6^Department of Mathematics, Vanderbilt University, Nashville, TN, United States

**Keywords:** visual transduction, cyclic GMP, rod outer segment, retina, single cell recording, mathematical modeling, salamander, toad

## Abstract

Accurate photon counting requires that rods generate highly amplified, reproducible single photon responses (SPRs). The SPR is generated within the rod outer segment (ROS), a multilayered structure built from membranous disks that house rhodopsin. Photoisomerization of rhodopsin at the disk rim causes a local depletion of cGMP that closes ion channels in the plasmalemma located nearby with relative rapidity. In contrast, a photoisomerization at the disk center, distant from the plasmalemma, has a delayed impact on the ion channels due to the time required for cGMP redistribution. Radial differences should be greatest in large diameter rods. By affecting membrane guanylate cyclase activity, bicarbonate could impact spatial inhomogeneity in cGMP content. It was previously known that in the absence of bicarbonate, SPRs are larger and faster at the base of a toad ROS (where the ROS attaches to the rest of the cell) than at the distal tip. Given that bicarbonate enters the ROS at the base and diffuses to the tip and that it expedites flash response recovery, there should be an axial concentration gradient for bicarbonate that would accentuate the base-to-tip SPR differences. Seeking to understand how ROS geometry and bicarbonate affect SPR variability, we used mathematical modeling and made electrophysiological recordings of single rods. Modeling predicted and our experiments confirmed minor radial SPR variability in large diameter, salamander rods that was essentially unchanged by bicarbonate. SPRs elicited at the base and tip of salamander rods were similar in the absence of bicarbonate, but when treated with 30 mM bicarbonate, SPRs at the base became slightly faster than those at the tip, verifying the existence of an axial gradient for bicarbonate. The differences were small and unlikely to undermine visual signaling. However, in toad rods with longer ROSs, bicarbonate somehow suppressed the substantial, axial SPR variability that is naturally present in the absence of bicarbonate. Modeling suggested that the axial gradient of bicarbonate might dampen the primary phototransduction cascade at the base of the ROS. This novel effect of bicarbonate solves a mystery as to how toad vision is able to function effectively in extremely dim light.

## Introduction

Rod photoreceptors in the vertebrate retina convert photons into electrical signals to provide for vision in dim light. The main cell body, or inner segment, extends a specialized cilium, called the rod outer segment (ROS), that is stacked with about a thousand disks whose membranes contain rhodopsin. In order for rods to accurately encode photons, they must generate highly amplified and reproducible single photon responses (SPRs). Wide fluctuations in SPR amplitude and shape would not allow for the overall response to increase linearly with the number of coincident photons and information about the timing of photon absorption would be degraded. Nevertheless, a cumulative body of evidence indicates numerous sources of variability affecting the peak and recovery phases of the SPR. One important source of variability arises from randomness in the timing of R* inactivation; slower shutoff of R* results in larger, more prolonged SPRs with a delayed time to peak (Rieke and Baylor, 1998; Whitlock and Lamb, 1999; Caruso et al., 2010). This source of variability does not appear to be prohibitive because rods manage to achieve a standard deviation for the SPR amplitude that is ~0.2 of the mean (Baylor et al., 1979b; Rieke and Baylor, 1998; Whitlock and Lamb, 1999).

A second source of variability arises from randomness in the location within the outer segment of the rhodopsin photoisomerization. Geometry and structural properties of the ROS affect the radial and longitudinal diffusion of the second messengers, cGMP and Ca^2+^, that influences the kinetics and amplitude of the SPR (Caruso et al., 2006; Bisegna et al., 2008). Mathematical modeling indicates that the spatiotemporal pattern of cGMP depletion in the intradiskal space depends upon the radial location of photoisomerization on a disk (Caruso et al., 2020). No differences were discerned in toad rods (Lamb et al., 1981), but this source of variability should increase with ROS diameter and experimental determinations in rods with larger outer segment diameters have yet to be reported. Inhomogeneity in cGMP levels over time and space caused by random PDE activations adds further variability to the early, rising phase of the SPR. However, as the response to R* grows, the impact of this source of variability diminishes as more PDE*s are recruited across the disk surface and the depletion of cGMP spreads over a greater volume (Caruso et al., 2020).

Randomness in the axial position of the rhodopsin photoisomerization could also generate SPR variability. In frog (*Xenopus laevis*) and in toad (*Rhinella marina,* formerly named *Bufo marinus*), SPRs elicited at the base of the ROS (nearest the inner segment) are considerably larger and faster than those elicited at the tip in the absence of added bicarbonate (Baylor et al., 1979a; Lamb et al., 1981; Schnapf, 1983; Mazzolini et al., 2015). Although the basis of these differences is not understood, hindrance of axial diffusion within the ROS by the stacked disks (e.g., Olson and Pugh, 1993) make it likely that there are axial gradients of ions and cascade components that alter phototransduction as a function of distance from the inner segment. Novel findings indicate Ca^2+^ levels in a dark adapted ROS rise as a function of distance from the base (Li et al., 2020). Because Ca^2+^ is a secondary messenger of phototransduction, such a gradient should cause peak amplitude as well as kinetics of the SPR to differ at the base and tip.

Bicarbonate is abundant and ubiquitous in the body; it is essential for pH regulation and it provides a means for the disposal of CO_2_, a metabolic waste product. In addition, bicarbonate increases dark current and accelerates flash response kinetics in vertebrate rods by enhancing the action of membrane guanylate cyclases that replenish cGMP after a photon response (Donner et al., 1990; Duda et al., 2015) and potentially, by altering intracellular pH (e.g., Liebman et al., 1984). Bicarbonate is taken up at the rod synapse, after which it moves to the ROS, where it is extruded by an anion exchanger (Koskelainen et al., 1994; Duda et al., 2016; Makino et al., 2019). If the movement is by passive diffusion, then there must be a higher concentration of bicarbonate at the ROS base than at the distal tip. The base vs. tip differences in the SPR of frogs and toads would already appear to be problematic for vision in dim light, and any substantive axial gradient of bicarbonate would accentuate those differences. A bicarbonate gradient might generate SPR variability in species that have shorter ROS length. In large diameter rods, there might even be a radial concentration gradient of bicarbonate. At the present time, nothing is known about the contribution of bicarbonate to SPR variability.

Here, we studied how SPR variability is affected by ROS dimensions and by an axial bicarbonate gradient, using a fully space-resolved, biophysical model of rod phototransduction and electrical recordings of single rods. The study revealed surprising differences in the effect of bicarbonate on SPR variability in the rods of two amphibian species: tiger salamanders (*Ambystoma tigrinum*) and toads (*Rhinella marina*).

## Materials and methods

### Animals

Larval tiger salamanders (*Ambystoma tigrinum,* Wadelco, Corpus Christi, TX), approximately 6–10 inches in length, were kept at 12°C and fed redworms twice a week. Cane toads (*Rhinella marina,* formerly named *Bufo marinus*, Backwater Reptiles, Rocklin, CA), 4–6 inches in length, were kept at 21–25°C and fed crickets twice a week. Similar numbers of male and female salamanders were used; sex of the toads was not determined. All animal care and use conformed to the Association for Research in Vision and Ophthalmology Statement for the Use of Animals in Ophthalmic and Vision Research and to a protocol approved by the Institutional Animal Care and Use Committee. For physiological experiments, retinas from animals that were dark adapted overnight were isolated under infrared illumination following euthanasia and stored in Ringer’s or in MOPS-buffered Ringer’s solution, on ice. Ringer’s solution contained (mM): NaCl, 108; KCl, 2.5; MgCl_2_, 1; CaCl_2_, 1.5; HEPES, 10; EDTA, 0.02; glucose, 10; bovine serum albumin (Fraction V, A-3059, Sigma), 7.4e-4; pH 7.6. MOPS-buffered Ringer’s contained (mM): NaCl, 58; KCl, 2.5; MgCl_2_, 1; CaCl_2_, 1.5; HEPES, 5; EDTA, 0.02, glucose, 10; bovine serum albumin, 7.4e-4; MOPS, 55; pH 7.6.

### Electrical recordings

**Figure 1:**
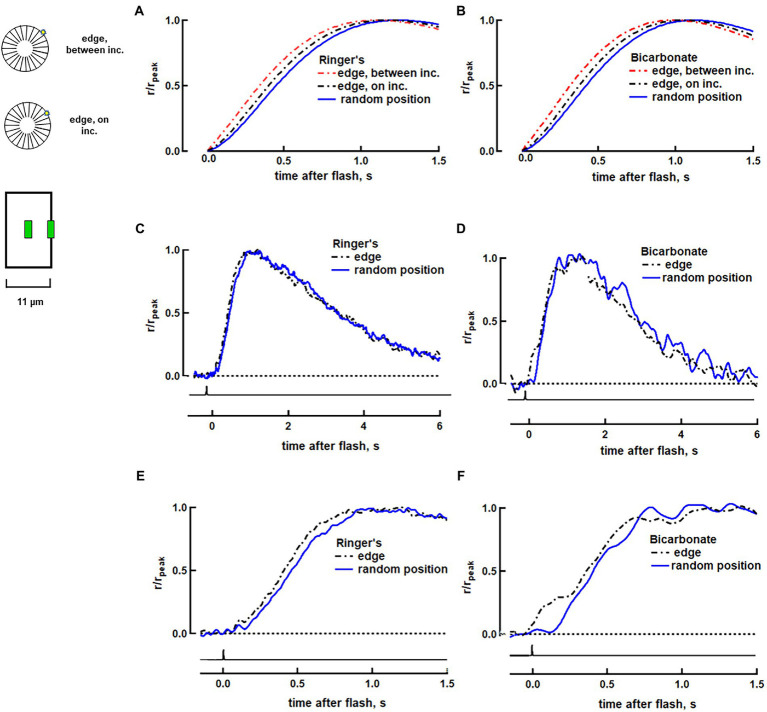
Faster rising phase with photoisomerization at the disk edge in a salamander rod. **(A)** Variability arising from R* position on a disk surface predicted by a biophysical model for rod phototransduction in Ringer’s. The three traces show the response normalized to peak current for R* located: at random radial positions (averaged), at the disk rim between incisures or at the disk rim adjacent to an incisure. The simulations were deterministic with the spread of transducin/PDE activation across the disk following the diffusion of heat on a surface and with R* and transducin/PDE activities shutting off over exponential time courses. The simulations did not fully reproduce the effective time; although the model incorporated diffusional delays, it did not include processing times, e.g., for the creation of R*, T* and PDE* or for the CNG channel to respond to the fall in cGMP. **(B)** Simulations in bicarbonate. Guanylate cyclase activity at high Ca^2+^ was increased by 7% and activity at low Ca^2+^ was increased by 100% to produce the 13% increase in dark current and the 16.5% reduction of time constant, τ, that was observed experimentally with bicarbonate (See full list of parameters in Supplementary Table S1). Responses to 25 to 70 dim flash trials were averaged and the mean was then computed for 11 rods in Ringer’s **(C,E)** and for 9 rods in 30 mM bicarbonate **(D,F)**. Traces were corrected for the 21 ms delay introduced by low pass filtering. Inset: Upper, visualization of photoisomerization position between and on incisures. OS represents in horizontal section. Lower, for single cell recordings, flashes were presented as a thin slit (green) centered along the length of the ROS and positioned either in the middle of the ROS or at an edge.

Shredded pieces of dark-adapted retina were placed in a recording chamber under infrared light and perfused continuously with Ringer’s solution, MOPS-buffered Ringer’s or Ringer’s solution containing bicarbonate at room temperature, 19–22°C. Photocurrent responses to flashes were recorded from single rods using the suction electrode technique with outer segment inside (ROS-in) the pipette, except for a few preliminary salamander experiments in Ringer’s, in which the inner segment was in the pipette (Baylor et al., 1979a; Makino et al., 2019). The pipette was filled with Ringer’s or with MOPS-buffered Ringer’s, pH 7.6, without albumin. Recordings were made with a current-to-voltage converter (Axopatch 200A, Axon Instruments, Foster City, CA), low-pass filtered at 20 Hz (−3 dB) with an 8-pole Bessel filter (Frequency Devices, Haverhill, MA) and digitized online at 400 Hz (Patchmaster v2x53, Heka, Holliston, MA). Traces were not adjusted for the delay introduced by low-pass filtering except in Figures 1C–F, where the recorded traces were offset by −21 ms. The recordings shown in the figures were subjected to additional digital filtering at 6.5 Hz (Igor Pro v7.02, Wavemetrics, Inc., Lake Oswego, OR). Flash duration for full field flashes was 21–22 ms. Salamander retinas contain two spectral types of rods, green-sensitive rods and blue-sensitive rods. Spectral type was determined by comparing the response amplitudes to flashes of similar intensity at 435 nm and 500 nm. All results were from green-sensitive rods.

**Figure 2:**
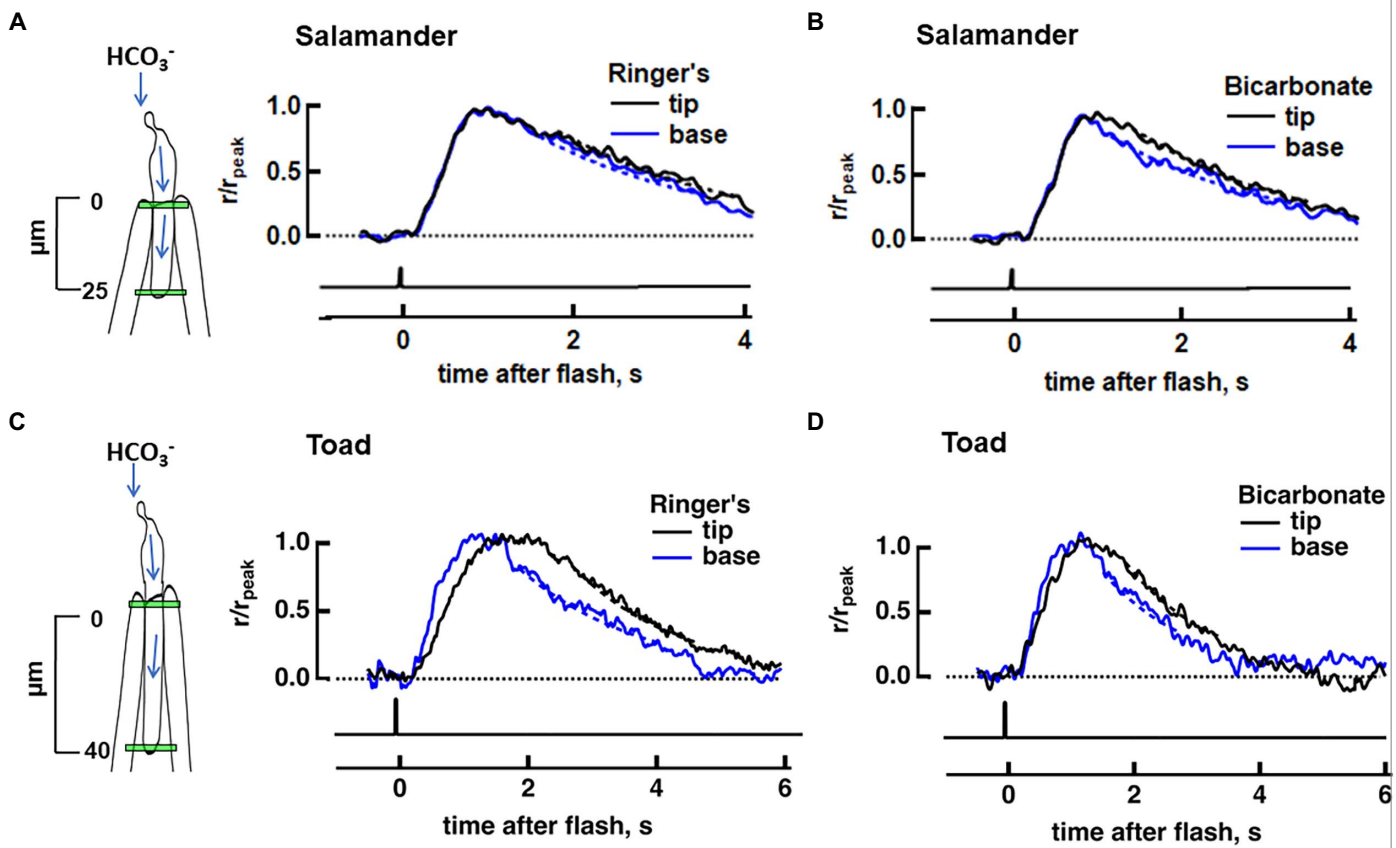
Effect of bicarbonate on the axial difference in SPR between base and tip in salamander and toad rods. Dim flash responses were normalized to their respective peak amplitudes. Dashed lines depict the exponential fits of the recovery phase. **(A)** No base vs. tip difference in the normalized, averaged dim flash responses in Ringer’s for eight salamander rods (4 cells treated with 30 mM bicarbonate and 4 cells treated with 50 mM bicarbonate). **(B)** Dim flash response at the base was faster than that at the tip during perfusion with bicarbonate for the same cells as in **(A)**. **(C)** Faster average dim flash response at the base, compared to the tip, in 11 toad rods in Ringer’s (9 cells treated with 30 mM bicarbonate and 2 cells treated with 50 mM bicarbonate). **(D)** Attenuated axial differences between base and tip photoresponse kinetics in bicarbonate for the same cells as in **(C)**. Inset: locations of slit illumination (green) during ROS-in recording. Blue arrows show the path for the intracellular diffusion of bicarbonate; it enters the rod at the synapse and moves to the outer segment, where it is extruded.

**Figure 3:**
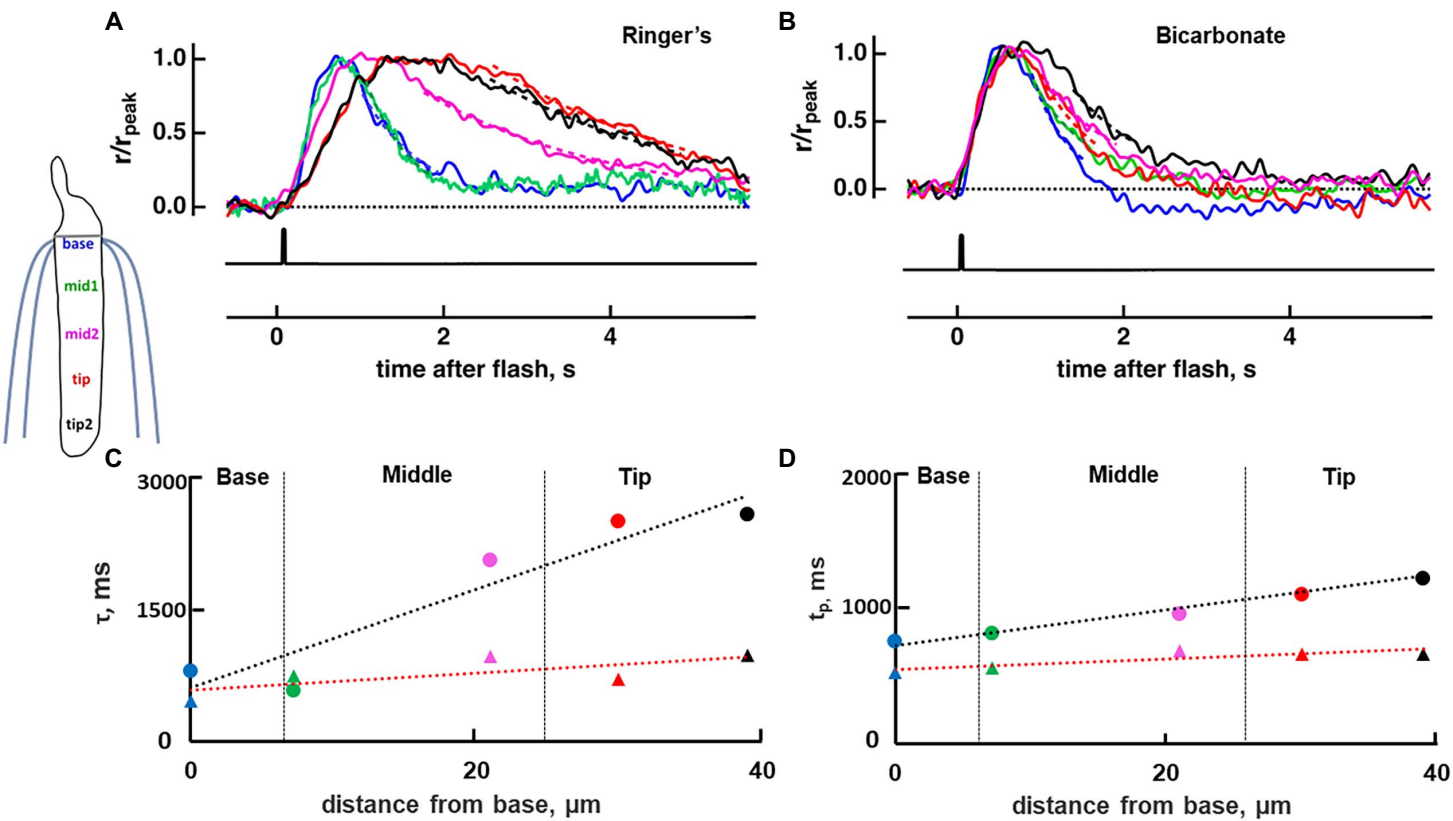
Diminished base to tip gradient in rising and recovery phase of a toad rod upon bath perfusion with 30 mM bicarbonate. Normalized dim flash response recorded in Ringer’s **(A)**, average of pretreatment and washed responses, or in bicarbonate **(B)** with a slit that was positioned at one of five locations along the ROS. Dashed lines show exponential fits to the recovery phases of the responses, from 80 to 20% r_peak_. **(C)** The profile of the response recovery time constants (τ) from the cell in **(A,B)**. Ringer’s (circles): slope = 56.8 ms/μm, r_Pearson_ = 0.94, *p* = 0.016; bicarbonate (triangles): slope = 9.858 ms/μm, r_Pearso_*n* = 0.74_,_ not significant. Dashed lines represent the linear regression, black for Ringer’s and red for bicarbonate. **(D)** The profile of the time to peak (t_p_), same labeling as in **(C)**. Ringer’s: slope = 17.10 ms/μm, r_Pearson_ = 0.99, *p* = 0.002; bicarbonate: slope = 8.66 ms/μm, r_Pearson_ = 0.98, *p* = 0.002; Colors in panels **(A–D)** correspond to positions shown in the inset. Inset: location of slit illumination during ROS-in recording.

To explore how location of the photoisomerization within the ROS affected the photon response, we passed flashes at 500 nm through one of two slit configurations. In one set of experiments, a slit that was 4 μm in length and less than 1 μm in width at the plane of the preparation was oriented parallel to the long axis of the ROS and located halfway between base and tip either at the edge of the ROS or at its center (Figure 1, inset). In a second set of experiments, the slit was positioned perpendicular to the ROS at various distances from the inner segment (Figure 2, inset; Figure 3, inset). Flash duration was 1–1.5 ms for both slit configurations. Flash response kinetics were determined for responses whose amplitudes were less than 0.25 of the maximum for full field flashes and less than 0.15 of the maximum for slit experiments.

Two concentrations of bicarbonate were tested. For the initial experiments with the 50 mM concentration, HCO_3_^−^ replaced an equimolar amount of MOPS in the MOPS-buffered Ringer’s. In later experiments with the 30 mM concentration, HCO_3_^−^ replaced Cl^−^ in the Ringer’s that did not contain MOPS. The solutions were not bubbled with O_2_/CO_2_ but were kept in covered reservoirs. Nevertheless, pH sometimes changed over a time scale of hours, so pH was measured after each recording session. A working range of 7.5 to 7.8 was deemed acceptable. Bath perfusion with Ringer’s containing bicarbonate prompted dark current to change over the subsequent 10 to 15 min and responses were typically measured more than 20 min after the switch.

### Biophysical space-resolved model

The fully space-resolved model of phototransduction along with the parameter values used to simulate the SPR in rods with incisures was described in (Bisegna et al., 2008; Caruso et al., 2010, 2020). Some parameters were adjusted in the present study for the effects of bicarbonate or for the structural differences in salamander and toad rods (see Supplementary Tables S1, S2). By rigorously incorporating the mathematical theories of homogenization and concentration of capacity, phototransduction in the ROS was modeled by a novel system of diffusion equations whose coefficients expressed the effects of the ROS’s small scale geometries (e.g., interdiskal and diskal thicknesses) which enabled the domain geometry, itself, to become greatly simplified for numerical simulation: the ROS interior volume became a cylinder, the outer shell became a cylindrical boundary, the disk where photoisomerization occurred became a horizontal cross-section, and the incisures became vertical cross-sections (i.e., rectangles) with one for each incisure. A finite element formulation of the homogenized model was used for the numerical simulations. In particular, the activated disk was discretized into triangular elements (three nodes for each triangle), the interior volume into prismatic elements with triangular bases (six nodes for each prism) and the outer shell and the incisures into rectangular elements (four nodes for each rectangle), which coincided with the faces of the prisms in the interior volume lying on these surfaces. Bilinear shape functions for the rectangles and prisms and linear shape functions for the triangles were used for interpolating the nodal unknowns inside the discretization elements. The standard iso-parametric map was employed for computation and for evaluating the mass and stiffness matrices relevant to the discretized problem. Finally, the Wilson-theta method, a finite-difference scheme that requires an iterative procedure due to the presence of nonlinear forcing terms in the model, was adopted for the time integration.

The formulation was implemented in MATLAB in a very general manner, allowing for: deterministic or stochastic simulations, single or multiple activations, the presence of incisures of any number and size, and the testing of different hypotheses concerning activation biochemistry and cascade components. Disk diameter was set to 11 μm for salamander, the approximate size of the rods that were recorded. For bicarbonate simulations, the guanylate cyclase minimum rate of cGMP synthesis at high [Ca^2+^] was raised by 13% and maximal rate at low [Ca^2+^] was raised by 100% to increase dark current by 13.0% and to reduce the time constant for the exponential recovery of the photon response by 16.5%, to match observations from physiological recordings. To model experiments with the slit positioned at the edge of the ROS next to an incisure or halfway between two adjacent incisures, the simulations were deterministic in that the position of the photoisomerization was chosen and inactivation of R* was given by the solution of a continuous time Markov chain (Caruso et al., 2010). To model experiments with the slit in the middle of the ROS, 100 stochastic simulations were carried out, with the R* positioned randomly at different distances from the disk center, and an average of the obtained responses was then computed.

### Experimental design and statistical analyses

Paired t-tests were used to assess whether a treatment or shift in slit position changed a flash response parameter (Excel version 2016, Microsoft, Redmond, WA). In cases for which there were few cells, statistical evaluations were made with a Wilcoxon signed-rank test for matched pairs performed by Social Science Statistics (n.d.).^1^ For this test, if 5 ≤ n ≤ 10, the *Z*-value was used to calculate the precise value for *p*.

Curve fittings were carried out using Igor Pro. Linear fits of the rising phases of dim flash responses were calculated from 20 to 60% r_peak_, using cells for which r_Pearson_^2^ > 0.8, to characterize the rising phase trajectory. Dim flash response recovery was analyzed by fitting an exponential function, r = A*exp(−t/τ), to the falling phase from 80 to 20% r_peak_. The per cent change was calculated for each cell individually as 100 x parameter for condition 1 divided by the parameter for condition 2, and then averaged across cells. For the effects of bicarbonate, bicarbonate treatment was condition 1 and the average of pretreatment with Ringer’s and Ringer’s wash was condition 2.

Single photon responses were stimulated with 30–100 dim flashes. Responses to single photoisomerizations were interspersed randomly amongst failures and multiple photoisomerizations. Responses from individual trials were fitted with a seventh-degree polynomial equation constructed from the mean response of that rod and the obtained histogram (bin width = 0.1 pA) was fitted with the equation (Baylor et al., 1979b):


(1) $$\begin{array}{l} p\left(\mathrm{r|}\sigma_{0},\;m,\;\sigma_{1},\;a\right)=\overset{\infty }{\underset{k=0}{\sum }}\frac{e^{-m}m^{k}}{k!}\;\frac{1}{{\left(2\pi \left(\sigma_{0}^{2}+k\sigma_{1}^{2}\right)\right)}^{0.5}}\\ \exp\left(-\frac{{\left(r-ka\right)}^{2}}{2\left(\sigma_{0}^{2}+k\sigma_{1}^{2}\right)}\right) \end{array}$$

where p(r|$\sigma_{0},m,\sigma_{1},a$) is the probability density of the response with amplitude in the range r to r + *d*r when conditioned on fixed parameter values ($\sigma_{0}$, m, $\sigma_{1}$, and a), k is the specified number of photoisomerizations per trial, m is the mean number of photoisomerizations per trial, a is the mean SPR amplitude, $\sigma_{0}$ is variance of background noise and $\sigma_{1}$ is response variance. Responses for different trials were taken as independent. Goodness of fit was evaluated with a Kolmogorov–Smirnov (KS) test (DeGroot, 1986) on the observed, experimental data when response amplitudes were drawn from eq. 1 and the parameters $\sigma_{0}$, m, $\sigma_{1}$, a were fixed at the values obtained from Igor Pro. We note that to sample eq. 1, it was sufficient to sample the joint distribution for the number of detected photons and response amplitude, first drawing k according to its marginal Poisson distribution and then drawing a response amplitude conditional on k according to its corresponding normal distribution (Robert and Casella, 2004). Then the marginal value of the response amplitude could be taken. A *p* < 0.05 was considered to indicate a statistically significant disagreement between the data and the predicted distribution of eq. 1. Since 30–100 trials may have been too few for convergence of the KS statistic to its asymptotic distribution, *p*-values were estimated by a Monte-Carlo scheme: under each set of experimental conditions, the corresponding density from eq. 1 was independently sampled as many times as there were experimental data points. This resulted in a sample of the KS statistic by then computing the maximum absolute difference between the obtained sample distribution function and the theoretical distribution function fitted by Igor Pro when evaluated across the observed experimental data points. This process was independently repeated 100,000 times resulting in 100,000 Monte-Carlo KS samples. The value of *p* was estimated by the fraction of samples with KS values at or worse than the value presented by the data. This procedure was independently performed 3 times to give 3 independent estimates of the *p*-values (see Supplementary Table S3). In computations, the infinite sum in eq. 1 was truncated to ensure an error term <1e-6.

## Results

### Faster SPR time to peak with photoisomerization at the disk edge

Our fully space-resolved biophysical model of rod phototransduction was used to predict how locality of rhodopsin photoisomerization on the surface of a salamander disk would affect SPR kinetics. SPRs were simulated in a rod that was 11 μm in diameter, with and without bicarbonate (see parameters in Supplementary Table S1). Twenty-three radial incisures were distributed evenly around the perimeter of each of its disks (Figure 1, upper inset). The middle position of the slit on the ROS was simulated with 100 stochastic trials, in which the photoisomerization was positioned at random distances from the disk rim. Positioning the slit on the ROS edge was simulated with two trials capturing the extreme possibilities, one for a photoisomerization bordering an incisure and one for a photoisomerization located halfway between neighboring incisures. It is emphasized that other than the location of the photoisomerization and changes associated with the presence of bicarbonate, all other parameters for ROS structure and for the cascade were invariant in these simulations. For both photoisomerization locations at the ROS edge, next to and between incisures, the responses were faster than the average response for random photoisomerization positions in terms of slope of the rising phase (by 10–14%) and time to peak of the response (by 60–70 ms) (Figure 1A). Given that bicarbonate raises cGMP levels in darkness and accelerates flash response kinetics by stimulating membrane guanylate cyclase activity (Duda et al., 2015), we wanted to explore how it would impact SPR differences due to radial position of the photoisomerization. As a first pass, bicarbonate concentration was assumed to be radially homogeneous. Simulations yielded SPR recovery time constants that were faster in bicarbonate than in Ringer’s, but similar for edge and random positions. In addition, the differences in times to peak between edge and random positions were preserved in bicarbonate (Figure 1B).

Experimental determinations were made by recording from large salamander rods whose outer segments were 9.5–12 μm in diameter. In previous studies, 25 mM or 50 mM bicarbonate replaced equimolar amounts of MOPS and Cl^−^ concentrations were low (Duda et al., 2015; Makino et al., 2019). To more closely approximate physiological levels of Cl^−^ in this study, the Ringer’s solution was prepared without MOPS, and bicarbonate when present, substituted for an equimolar amount of Cl^−^. In control, ROS-in experiments carried out in the absence of bicarbonate, we observed no differences with full field flash stimulation in: dark current, sensitivity to flashes, or dim flash kinetics upon switching between Ringer’s solution and “Ringer’s” containing 50 mM MOPS in place of an equimolar amount of Cl^−^ (*n* = 3, results not shown). In 4 out of 4 rods attached to a piece of tissue, dark current increased from 25 ± 3 pA to 28 ± 3 pA, integration time of the dim flash response decreased from 2,230 ± 260 ms to 1,800 ± 110 ms indicative of a faster flash response recovery, and i_0.5_ values increased from 10 ± 3 photons/μm^2^ to 12 ± 4 photons/μm^2^ indicating no change in relative sensitivity to flashes, upon treatment with 30 mM bicarbonate (MOPS was absent from both solutions). All bicarbonate-induced changes were reversible. Time to peak of the dim flash response was not altered. These effects were comparable to results obtained with 50 mM bicarbonate in experiments with MOPS-buffered Ringer’s (Makino et al., 2019), suggesting that for salamander rods, bicarbonate exerted a more potent effect in normal Ringer’s (Table 1). All subsequent recordings of salamander rods were made with Ringer’s lacking MOPS.

**Table 1:** Changes in photoresponse parameters upon bath perfusion with bicarbonate.

Species, Ringer’s vs. Bicarbonate	*r*_max_	*T_i_*	*t_p_*	*i*_0.5_	*n*
Salamander, Ringer’s vs. 30 mM bicarbonate	1.12 ± 0.04, p = 0.001	0.79 ± 0.04, *p* = 0.043	1.0 ± 0.1, ns	1.3 ± 0.1, *p* = 0.005	4
Salamander, 55 mM MOPS -buffered Ringer’s vs. 50 mM bicarbonate^a^	1.11 ± 0.02, *p* = 0.00013, *n* = 12	0.81 ± 0.06, *p* = 0.008, *n* = 12	1.00 ± 0.05, ns, *n* = 6	1.2 ± 0.1, *p* = 0.033, *n* = 10	–
Toad, Ringer’s vs. 30 mM bicarbonate	1.08 ± 0.02, *p* = 0.049	0.81 ± 0.05, *p* = 0.014	1.01 ± 0.03, ns	1.1 ± 0.1, ns	7
Toad, 55 mM MOPS- buffered Ringer’s vs. 50 mM bicarbonate	1.30 ± 0.06, *p* = 0.003	0.60 ± 0.08, *p* = 0.043	1.1 ± 0.1, ns	1.7 ± 0.2, *p* = 0.040	5

All cells were recorded with ROS-in and stimulated with full field flashes. Changes are expressed as ratios of the parameter in bicarbonate divided by that in Ringer’s or MOPS-buffered Ringer’s. *r*_max_, saturated photoresponse amplitude which provided a measure of dark current; *T_i_*, integration time of the dim flash response; *t_p_*, time to peak of the dim flash response; *i*_0.5_, flash strength at 500 nm producing a half-maximal flash response; *n*, number of cells. For the results of the present study, reversibility of bicarbonate treatment was not established for every parameter of every rod. Values given as mean ± SEM, value of *p*. *P*-values were from paired *t*-tests between parameter values given in the text (not ratios) in Ringer’s and bicarbonate. ns, not significant.

^a^from Makino et al. (2019).

A separate group of rods was then stimulated with slit illumination. The ROS, or in preliminary experiments, the inner segment, was pulled into a suction pipette and a tiny slit of light was presented side-on as a dim flash, the response to which had the same kinetics as the SPR. Placement of the slit near the edge of the ROS (Figure 1, lower inset) gave rise to photoisomerizations near the rim of the disk. With the slit in the middle of the ROS, photoisomerizations occurred at random radial distances from the disk rim. It was not possible to control proximity of the photoisomerization to an incisure at either slit location. Somewhat brighter flashes were often required with the slit at the edge, due to the reduced pathlength and because a portion of the slit was positioned past the boundary of the ROS to ensure that any photoisomerizations would be as close to the disk rim as possible. Responses peaked earlier for dim flashes at the disk edge (e.g., Figures 1C,E): *t*_p_ edge = 880 ± 80 ms, *t*_p_ middle = 1,010 ± 100 ms (mean ± SEM, *n* = 11, *p* = 0.002 from a paired t-test). No differences in response recovery time constant, τ, nor in integration time *T*_i_, the integral of the response divided by its peak amplitude, were detected (Figure 1C), which meant that radial location of photoisomerization did not noticeably affect the later phase of response recovery. These results were consistent with our modeling that showed a slightly faster average SPR for a photoisomerization at the edge of the disk compared to that for random radial positions (Figure 1A) and a larger discrepancy with the SPR for a photoisomerization in the disk center (see Caruso et al., 2020).

The differences between the responses to photoisomerizations at the disk edge vs. random locations were preserved upon treatment with 30 mM bicarbonate. Time to peak remained 16 ± 2% faster at the edge: *t*_p_ edge = 870 ± 70 ms, *t*_p_ middle = 1,020 ± 100 ms (*p* = 0.001). Rising phase slope (e.g., Figures 1D,F) and integration time remained similar for both slit positions (Figure 1D); the main difference was in the shorter delay of onset for the responses at the edge. It was not possible to assess differences in SPR amplitude at the two slit positions in the presence or absence of bicarbonate, because of excessive drift in the baseline that likely arose from noise in the phototransduction cascade (Baylor et al., 1980; Vu et al., 1997). Our modeling and experimental results were thus consistent. We conclude that radial position of the photoisomerization introduced some variability to the SPR in large salamander rods and that variability was unchanged by bicarbonate.

### Axial SPR gradient generated by bicarbonate in salamander rods

In order to quantify the effect of an axial gradient of bicarbonate in the outer segment on SPR variability, we recorded flash responses from single salamander rods with ROS inside the pipette so that the synapse could access bicarbonate when it was added to the bath. First, we stimulated the rod in the absence of bicarbonate with dim flashes that passed through a tiny slit positioned either near the base or near the tip of the ROS in order to check for axial invariance of SPR kinetics. The distance between the two locations was ~25 μm. The average of each set of photoresponses was normalized to its respective peak, for comparison. Usually, a higher flash strength was required for the base, probably because a portion of the slit overlapped with inner segment and because some fraction of the light was scattered by the curvature at the end of the polished suction pipette. To check that the test flashes were dim enough and fell within the linear range, responses to at least two flash strengths were recorded at each position. Responses to the two weakest flash strengths varied only in amplitude, confirming linearity (e.g., Supplementary Figure S1) but as an extra precaution, further analysis was restricted to the responses to the dimmer of the two flashes. The largest mean ensemble response in any of the salamander rods for this dim slit stimulation was 0.8 pA.

No significant differences in kinetics were detected between dim flash responses at the two axial locations as assessed by time to peak or integration time in the absence of bicarbonate (Figure 2A). Rods were then perfused with bicarbonate for 20–30 min to allow conditions to reach a steady state, before we resumed the recording. An axial gradient of bicarbonate would accelerate responses at the base of the ROS more than at the tip. In fact, recovery did kick in sooner at the base with bicarbonate (Figure 2B) in every rod tested. For the analysis, we combined results from 4 cells treated with 30 mM bicarbonate and 4 cells treated with 50 mM bicarbonate, causing integration time to be 26 ± 5% shorter at the base: *T_i_* base = 1,746 ± 275 ms, *T_i_* tip = 2,241 ± 337 ms (*p* = 0.0058, Wilcoxon signed rank test for matched pairs). These experiments confirmed that there was an axial gradient of bicarbonate concentration in salamander ROSs with a higher concentration at the base, that gave rise to a faster SPR recovery at the base, compared to the tip.

### *Attenuated* axial differences in the SPR with bicarbonate in toad rods

**Figure 4:**
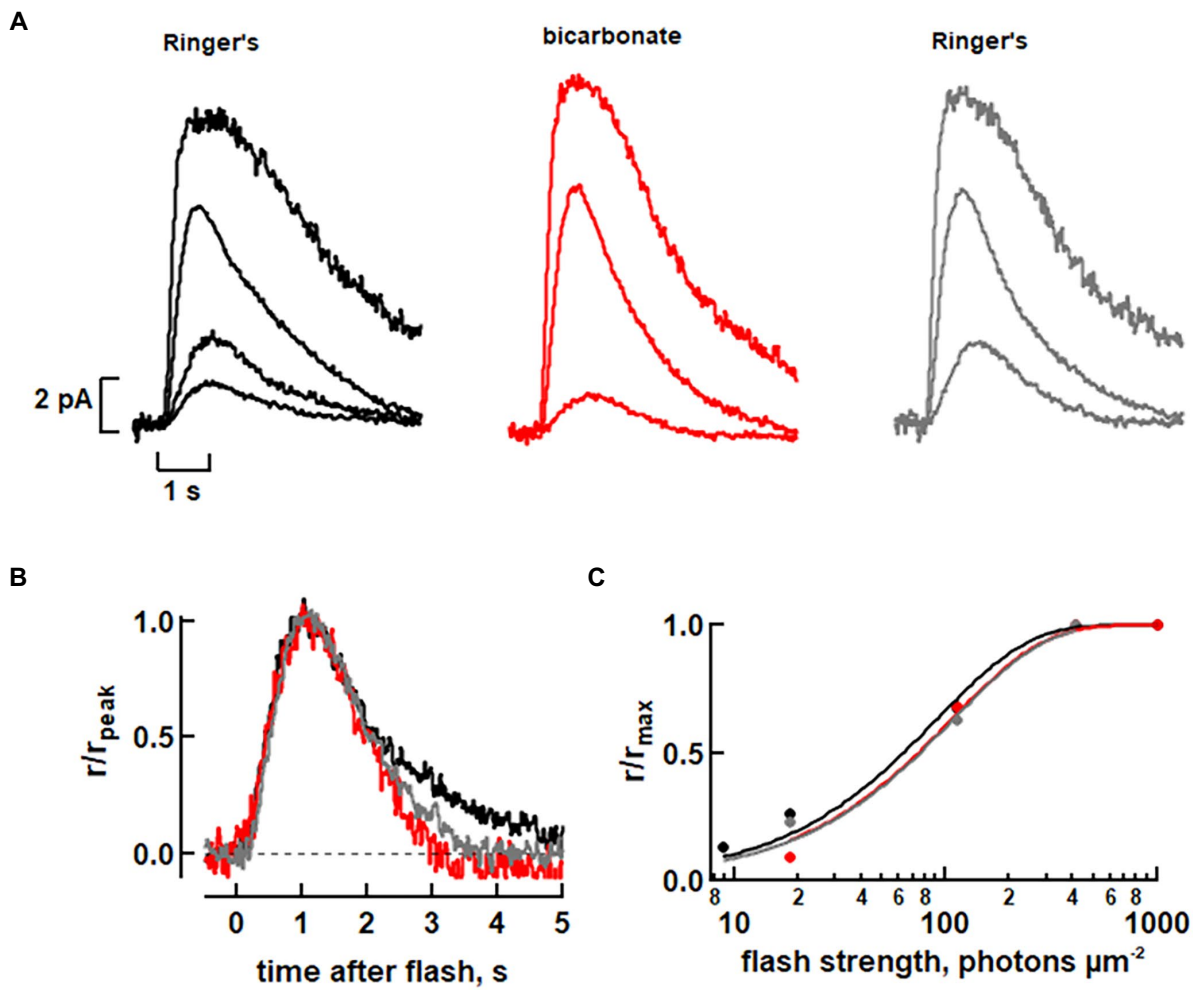
Faster flash response kinetics with 30 mM bicarbonate in a toad rod. **(A)** Rod attached to a clump of retinal cells was recorded with its ROS inside the pipette and stimulated with full field flashes at 500 nm. **(B)** Faster photon response recovery with bicarbonate. Dim flash responses from **A**, whose peak amplitudes were less than a fifth of the maximum, were scaled to their peak amplitudes. Integration time was ~19% less with bicarbonate. **(C)** Little change in relative sensitivity to flashes of the rod with bicarbonate. Results from **A** were fit with a saturating exponential function: r/r_max_ = 1 – exp(−ki) where k is a constant equal to ln(2)/i_0.5_ and i_0.5_ is the flash strength eliciting a half-maximal response.

We expected to observe even more axial variability in a toad rod since its ROS is twice as long and should support a more pronounced base to tip difference in bicarbonate. To ensure bicarbonate uptake, rods were recorded from pieces of retina. We first carried out background experiments on 5 toad rods perfused with MOPS-Ringer’s and stimulated with full field flashes to characterize the changes induced by bicarbonate under our recording conditions. Fifty mM bicarbonate increased dark current from 9.0 ± 0.4 pA before treatment to 11.0 ± 0.6 pA, reduced *T*_i_ from 2,620 ± 140 ms to 1,530 ± 80 ms, and lowered relative sensitivity with i_0.5_ changing from 89 ± 8 photons/μm^2^ to 163 ± 15 photons/μm^2^. Our toad rod responses to bicarbonate were consistent with those of salamander rods above and in previous studies (Duda et al., 2015; Makino et al., 2019), making toad rods suitable for further investigation of the effect of bicarbonate on SPR variability. A previous study reported a twofold reduction in time to peak and a fourfold decrease in flash sensitivity in 22 mM bicarbonate compared to HEPES-buffered Ringer’s (Lamb et al., 1981). Our results with higher concentrations of bicarbonate were less striking, perhaps due to differences in ion concentrations in the Ringer’s and to our use of MOPS. Since bicarbonate was more effective on salamander rods during perfusions with Ringer’s lacking the MOPS, similar experiments without MOPS were carried out on an additional 7 toad rods stimulated with full field flashes. Thirty mM bicarbonate increased dark current from 10.1 ± 0.6 pA in Ringer’s to 11.1 ± 0.7 pA and reduced the integration time of the dim flash response from 1,900 ± 70 ms in Ringer’s to 1,570 ± 110 ms, but did not change time to peak (Figures 4A,B), similar to results in salamander. Relative sensitivity was not changed by bicarbonate (Figure 4C), nor was flash sensitivity, defined as dim flash response amplitude divided by flash strength. Collected results from all of the salamander and toad rods upon treatment with bicarbonate in Ringer’s with and without MOPS, are summarized in Table 1.

For direct comparison to the results in salamander, slit experiments on toad rods were carried out in Ringer’s lacking MOPS. In the absence of bicarbonate, toad rods exhibit a natural difference in photon responses elicited at the base of the ROS compared to those elicited at the tip (Baylor et al., 1979a; Lamb et al., 1981; Schnapf, 1983; Mazzolini et al., 2015) that is not present in salamander rods (Figures 2A,C). Upon flashing 11 toad rods with narrow slits at two axial locations separated by ~40 μm, we also observed faster response kinetics at the base. Time to peak was shorter: 1,230 ± 140 ms at the base, 1,540 ± 130 ms at the tip (*p* = 0.005), recovery time constant was faster: 1.4 ± 0.2 s at the base, 2.7 ± 0.3 s at the tip (*n* = 10, *p* = 0.009), and integration time was briefer: 2,130 ± 210 ms at the base, 2,730 ± 170 ms at the tip (*n* = 10, *p* = 0.023). Acceleration of the time to peak of the response upon perfusion with bicarbonate was significant at both base (1,230 ± 140 ms in Ringer’s, 1070 ± 150 ms in bicarbonate, *n* = 11, *p* = 0.043) and tip (1,540 ± 130 ms in Ringer’s, 1,180 ± 130 ms in bicarbonate, *n* = 11, *p* = 0.003), but in marked contrast to salamander rods, the acceleration in toad rods was greater at the tip (*n* = 11, *p* = 0.019) so that the times to peak and the response recoveries at the two axial positions were no longer different, *p* = 0.116 (Figures 2C,D). The disparity was investigated in greater depth by linear regression of the dim flash response from 20 to 60% r_peak_ to ascertain the slope of the rising phase. There was a steeper slope for the rising phase (1.0 ± 0.1 pA/ms in Ringer’s, 1.3 ± 0.2 pA/ms in bicarbonate, *n* = 7, *p* = 0.025) at the tip of the toad ROS, upon perfusion with bicarbonate. At the base, the slope of the rising phase was not changed upon perfusion with bicarbonate (2.4 ± 0.4 pA/ms in Ringer’s, 2.0 ± 0.3 pA/ms in bicarbonate, *n* = 7, *p* = 0.296).

**Figure 5:**
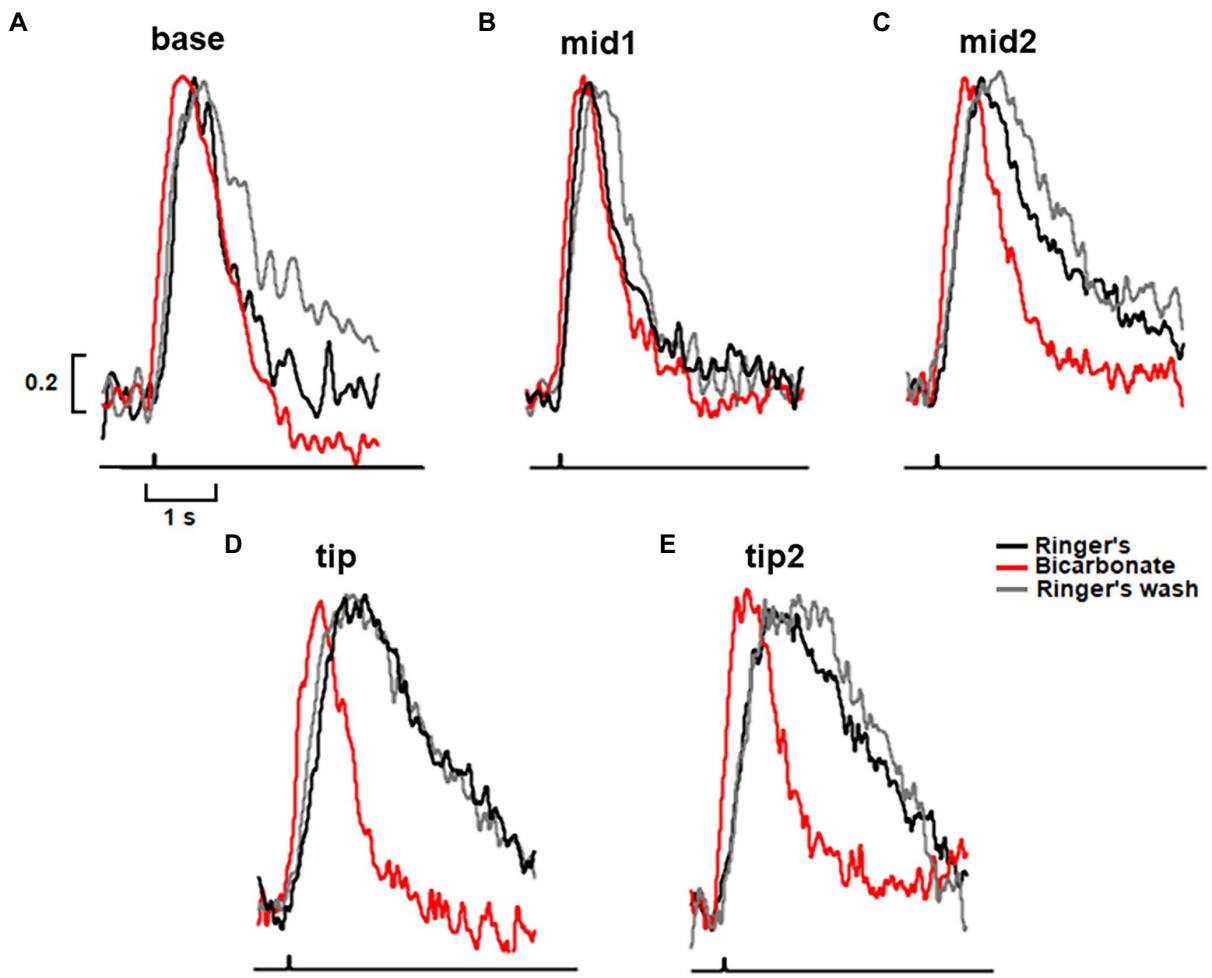
Greatest acceleration by bicarbonate of the rising and recovery phases of the dim flash response to flashes presented to the tip of the outer segment of a toad rod. **(A–E)** Traces from the same rod as in Figure 3. Dim flash responses were normalized to their peaks in Ringer’s and in bicarbonate with the slit positioned along the ROS, as shown in the inset to Figure 3. For Ringer’s: *t*_p_(base) = 740 ms, *t*_p_(mid1) = 750 ms, *t*_p_(mid2) = 820 ms, *t*_p_(tip) = 1130 ms, *t*_p_(tip2) = 1130 ms, τ(base) = -770 ms, τ(mid1) = 848 ms, τ(mid2) = 1670 ms, τ(tip) = 2220 ms, τ(tip2) = 2630 ms. For bicarbonate: *t*_p_(base) = 530 ms, *t*_p_(mid1) = 560 ms, *t*_p_(mid2) = 690 ms, *t*_p_(tip) = 670 ms, *t*_p_(tip2) = 670 ms, τ(base) = 460 ms, τ(mid1) = 750 ms, τ(mid2) = 960 ms, τ(tip) = 710 ms, τ(tip2) = 980 ms. For Ringer’s wash: *t*_p_(base) = 770 ms, *t*_p_(mid1) = 880 ms, *t*_p_(mid2) = 900 ms, *t*_p_(tip) = 1,070 ms, *t*_p_(tip2) = 1,430 ms, τ(base) = 1,270 ms, τ(mid1) = 820 ms, τ(mid2) = 1,720 ms, τ(tip) = 2,270 ms, τ(tip2) = 2,500 ms. Each trace is an average of 30–50 trials.

To map the axial gradient in rising and recovery phases, dim flash responses were recorded at 5 ROS locations in 6 toad rods. Results from one of these cells are shown in Figures 3, 5. For the analysis of all 6 rods, we combined results from 3 cells treated with 30 mM bicarbonate and 3 cells treated with 50 mM bicarbonate, because the parameters did not differ between the two groups. The τ for response recovery increased linearly with distance from the base (slope = 53 ± 23 ms/μm), but bicarbonate appeared to flatten the relationship (slope = 15 ± 13 ms/μm, *p* = 0.079) (e.g., Figure 3C). Time to peak in Ringer’s also increased linearly with distance from the base with a slope of 14.2 ± 0.9 ms/μm, but the slope was reduced 5.7-fold with bicarbonate, to 2.6 ± 2.1 ms/μm (*p* = 0.002) (e.g., Figure 3D). These results showed again that bicarbonate made the SPR more homogeneous by preferentially making responses at the tip faster (see Discussion).

**Figure 6:**
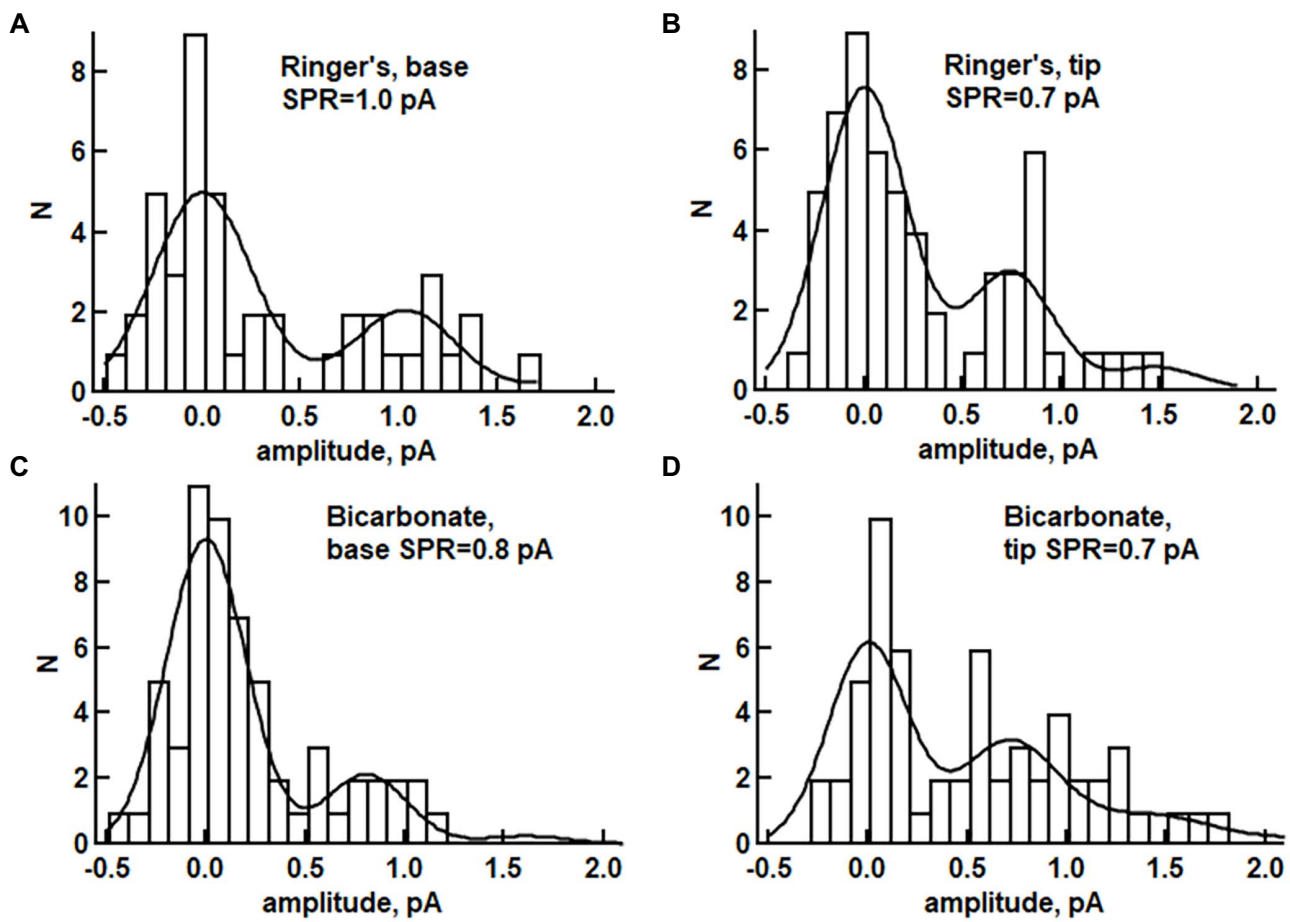
Histograms of response amplitudes for a toad rod exposed to dim flashes during perfusion without bicarbonate **(A,B)** or for a different rod perfused with 30 mM bicarbonate **(C,D)**. Slit illumination was positioned either at the base of the ROS (*n* = 48 for panel **A**, *n* = 60 trials for panel **C**) or at the tip (*n* = 61 for panel **B**, *n* = 60 trials for panel **D**). The distributions were fitted with eq. 1 as described in Materials and methods, then assessed with a Kolmogorov–Smirnov significance test for goodness of fit (*p*-values for **A–D**: 0.097, 0.563, 0.917, 0.339). **(A)** *p* < 0.05 was taken as evidence for statistically significant disagreement between the model prediction and the data, see, Supplementary Table S3; SPR is the mean response to a single photon.

Axial differences in SPR size in toad rods were assessed by plotting the amplitudes of dim flash responses in frequency histograms and fitting with a probability density function renormalized for frequency (see Materials and methods). In the absence of bicarbonate, the SPR at the base was 24 ± 7% larger than that from the tip (e.g., Figures 6A,B): base 0.88 ± 0.08 pA, tip 0.68 ± 0.08 pA (*n* = 6, *p* = 0.042), consistent with reports by others (Baylor et al., 1979a; Lamb et al., 1981; Schnapf, 1983; Mazzolini et al., 2015). With 30 mM bicarbonate, SPR amplitude between the base and tip of the ROS no longer differed (e.g., Figures 6C,D): base 0.61 ± 0.06 pA, tip 0.67 ± 0.06 pA (*n* = 10, *p* = 0.175). These bicarbonate-induced changes in toad rods contradicted our expectations for accentuated differences in the SPRs at the base and tip, due to an axial gradient for bicarbonate that would stimulate higher rates of cGMP synthesis at the base. Instead of making SPRs at the base and tip more disparate, bicarbonate functioned as a neuromodulator that *reduced* the axial variability of photon responses in toad rods.

### Modeling the base versus tip differences in toad rod SPRs

**Figure 7:**
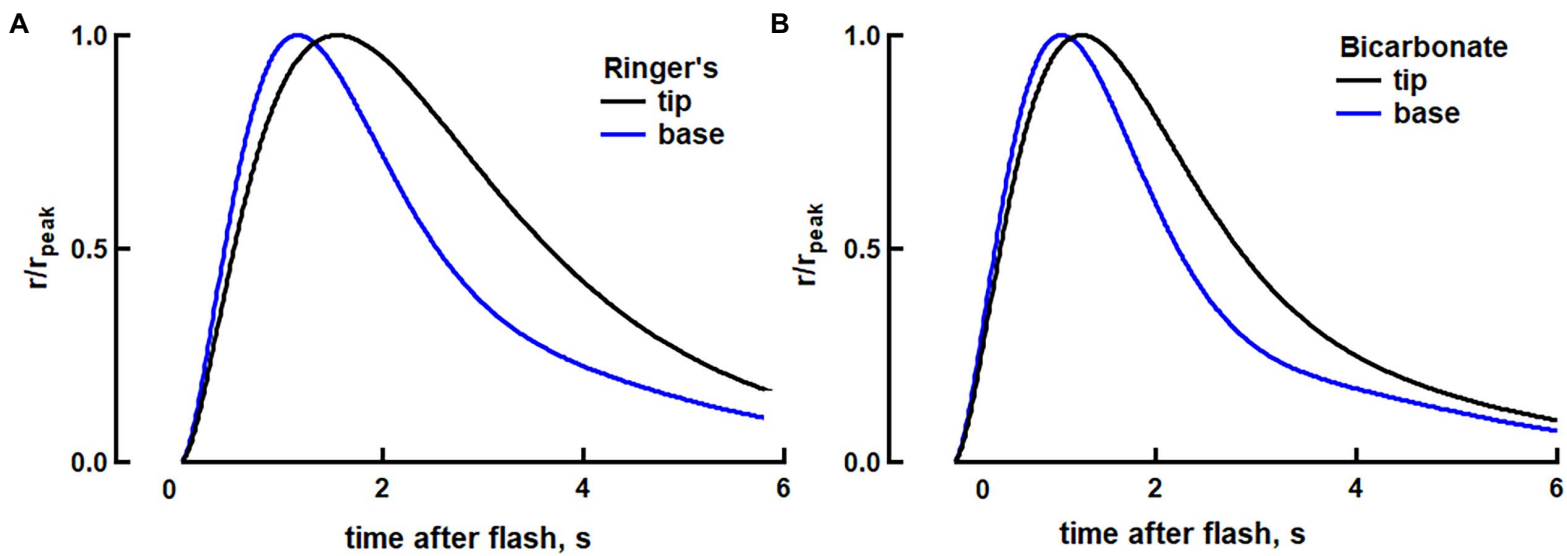
Modeling of the reduction in the kinetic differences between SPRs at the base vs. tip of a toad rod. **(A)** Modeling of faster dim flash response at the base, compared to the tip, in toad rods in Ringer’s (see text for a description of our approach of using separate rods to simulate base and tip). The two traces show photon responses normalized to their peaks at base and at the tip of the ROS. For the response at the tip, ν_RG_ was 48% lower and f_Ca_ was 56% higher, compared to the values for the base. **(B)** Attenuated axial differences in photoresponse kinetics in bicarbonate between the base and tip. At the base, guanylate cyclase activity at low Ca^2+^ was increased by 100% to increase circulating dark current by 8% and reduce integration time by 14%. At the tip, guanylate cyclase activity at low Ca^2+^ was increased by 30% and the fraction of the dark current carried by Ca^2+^ was decreased by 25%, with respect to Ringer’s, to produce a 9% increase in the circulating dark current and to reduce integration time by 16% to match the changes that were observed experimentally with bicarbonate. Finally, the rate of transducin activation by R* was decreased by 30% at the base in order for the rising phases at base and tip to more closely match (full list of parameters given in Supplementary Table S2).

The basis for the axial differences in SPR in toad rods in the absence of bicarbonate is not known. As a working hypothesis, we hypothesized that there might be higher transducin levels (Sokolov et al., 2002) and lower Ca^2+^ levels at the base (Li et al., 2020) than at the tip. We then used the fully space-resolved model to test whether SPRs at the base and tip would match more closely upon the addition of bicarbonate. Experimental determinations of axial concentration gradients of bicarbonate or cascade proteins in toad rods are not yet available, nor is there information on the axial diffusion of these substances. So, simulations were carried out in two theoretical rods for which second messengers and cascade proteins were homogeneously distributed throughout their ROSs (Figure 7). Rod-b was assigned concentrations of transducin, Ca^2+^ and bicarbonate that might be characteristic of the base and rod-t was assigned concentrations characteristic of the tip of a normal toad rod. Some other parameters for amphibian rods were adjusted within their reported ranges to obtain a closer fit to the traces in Figure 2C (Supplementary Table S2).

The effect of a lower transducin concentration at the tip was modeled in rod-t by decreasing the rate of transducin activation by R* by 48%. Higher Ca^2+^ at the tip could arise from Ca^2+^ release from disks, but we modeled it in rod-t as a 56% increase in the fraction of dark current carried by Ca^2+^, resulting in a 60% increase in the total Ca^2+^ in darkness. These changes were made so that the response in rod-b would have a rising slope that was 36.3% steeper, a time to peak that was 39.5% shorter, and a recovery time constant, τ, that was 307 ms faster compared to the responses in rod-t, for consistency with the base vs. tip differences in our toad rod recordings.

Taking into account that bicarbonate accelerates cGMP production and opens more channels, we increased the maximal rate of cGMP synthesis by 100% in rod-b. That change increased the dark current in rod-b by 8%. We assumed that the tip of a toad rod would have less bicarbonate than at the base, therefore we increased maximal rate of cGMP synthesis by 30% in rod-t. We also decreased by 25%, the fraction of dark current carried by Ca^2+^ with respect to Ringer’s for rod-t on the grounds that Ca^2+^ release from disks would make a reduced relative contribution to the cytosolic Ca^2+^ levels. The net effect was to increase dark current in rod-t by 9%. Consistent with the effects of bicarbonate on flash responses at the base and tip of toad rods in our electrophysiological recordings, bicarbonate sped up the SPRs in both rod-b and in rod-t but in order to reduce the kinetic differences in the normalized responses between rod-b and rod-t that were observed experimentally, it was necessary to impose an additional change. In our modeling, we reduced the rate of transducin activation by R* in rod-b by 30% when bicarbonate was present. Then the difference in time to peak was half that in Ringer’s and the difference in recovery time constant was reduced to 127 ms. The decrease in integration time with bicarbonate was 13% for rod-b and 16% for rod-t. Therefore, modeling suggested that bicarbonate could decrease SPR variability by attenuating the primary cascade at the base or by having the opposite effect at the tip in toad rods.

## Discussion

### Radial variability in the SPR of large diameter rods

Randomness in the site of rhodopsin photoisomerization on the disc surface could generate variability in the rising phase of the SPR because photoisomerization at the edge of a disk initiates a local depletion of cGMP near the plasma membrane that closes CNG channels after a brief delay. In contrast, the local fall in cGMP following photoisomerization at the disk center takes more time to impact the CNG channels, because the fall in cGMP must spread radially before the disturbance reaches the channels in the plasma membrane. Moreover, the cGMP depletion dissipates axially and tends to spread around the circumference of the ROS, effecting a smaller change in cGMP levels in a more symmetric annulus at the plasma membrane. The CNG channel has a Hill coefficient > 1, so fewer channels close. In the present study, the fully space-resolved, mathematical model of rod phototransduction predicted that the SPR at the disk edge would manifest with a shorter delay and steeper rate of rise to a larger amplitude than the SPR in the middle of the disk (Figures 1A,B; see also Caruso et al., 2020). The disparity would be greatest for a photoisomerization at the disk edge occurring halfway between adjacent incisures.

Experimentally, no differences were detected in the dim flash responses elicited with slit illumination positioned either at the ROS edge or centered on the ROS of a toad rod (Lamb et al., 1981). However, disparities in SPR amplitude and kinetics arising from the radial position of the photoisomerization increase with ROS diameter (Caruso et al., 2020), so it was important to check whether variability might be present in salamander, for which ROS diameter can be twice as large as in toad. Another consideration was that previous modeling indicated that a radially symmetric array of incisures reduces SPR variability by promoting axial diffusion of cGMP and Ca^2+^ within the cytosol (Caruso et al., 2006, 2011, 2020; Bisegna et al., 2008), but in reality, the incisures in salamander rods follow tortuous paths, are of unequal lengths, and are not always evenly spaced around the ROS perimeter (Mariani, 1986). Asymmetric partitioning of the disk membrane surface would restrict lateral diffusion of membrane proteins and cause variability in the number of PDE activated. Here again, the effect could be more significant in larger diameter disks. In our experiments, we were able to elicit photoisomerizations at the disk edge, but we were not able to restrict photoisomerizations to the disk center. Therefore, we could only compare SPRs at the disk edge to the average SPR elicited at random distances from the disk center, a limitation that would have diminished any differences. Nevertheless, SPRs at the disk edge were found to peak sooner than those elicited at other positions (Figure 1). Thus, locality of the photoisomerization on the disk surface does contribute to SPR variability in rods with large diameter ROSs. The magnitude of the difference conformed with predictions of our model, which incorporated radial incisures evenly spaced around the disk perimeter, suggesting that incisure asymmetry was not a major factor with regard to SPR variability.

Bicarbonate enters the ROS at the base and diffuses to the tip, but along the way, it is removed from the outer segment in exchange for chloride (Koskelainen et al., 1994; Duda et al., 2016; Makino et al., 2019) The location of the exchangers at the plasma membrane could affect SPR reproducibility by establishing a radial gradient of bicarbonate in the cytosol between the disks that affects the local rates of cGMP synthesis. As a starting point for modeling, bicarbonate levels were assumed to be radially homogeneous. The model predicted that axial differences in the SPR would still be present with bicarbonate and experimental observations were consistent, arguing against the existence of a large, radial bicarbonate gradient.

The “unreliable” rising phase of the SPR may be prohibitive soon after a very dim flash, but CV due to randomness in position of photoisomerization drops to very low levels after several hundred ms. Bicarbonate does not change this source of variability, but regardless, randomness in the radial location of the photoisomerization does not appear to constitute a major source of SPR variability, even in the large rods of salamander.

### ROS-length dependent effects of bicarbonate on the axial variability of the SPR

Bicarbonate enters the ROS at the base, so immediately after switching the perfusion to bicarbonate, there must be a higher concentration of bicarbonate at the ROS base than at the distal tip. We wanted to find out whether the axial concentration gradient dissipates significantly with continued perfusion. Our experiments on salamander clearly indicated that the axial concentration gradient during long perfusions with bicarbonate was great enough to be detected; flash responses at the base and tip were the same in the absence of bicarbonate, but recovery was faster at the base than at the tip with bicarbonate (Figures 2A,B). Toad rods are longer than salamander rods, so the hypothesis was that the bicarbonate-induced base to tip differences in the SPR would be more pronounced than in salamander. Furthermore, there are already differences in SPR amplitude and kinetics at the base and tip of toad ROSs in the absence of bicarbonate, that were expected to be accentuated by bicarbonate. To our surprise, bicarbonate actually reduced the base to tip SPR differences (Figures 2C,D, 3, 5, 6).

The basis for the axial variability in SPR existing in the absence of bicarbonate is not yet known. However, the stack of membranous disks within the elongated ROS creates a barrier for the axial diffusion of substances within the cytoplasm. The occurrence of axial gradients of second messengers and cascade components in the absence of bicarbonate could cause SPRs from the tip of a frog or toad ROS to rise more slowly to a smaller amplitude and then take longer to recover, than those from the base (Baylor et al., 1979a; Lamb et al., 1981; Schnapf, 1983; Mazzolini et al., 2015). Our results on toad rods are consistent with these observations (Figures 2C, 3A,C,D, 5, 6A,B), whereas in salamander rods in Ringer’s without bicarbonate, we did not detect this phenomenon (Figure 2A). A simple explanation is that salamander ROSs, being half as long as those of a toad or frog (Mariani, 1986; Nickell et al., 2007; Lu et al., 2018), did not support axial gradients of second messengers or cascade components that were steep enough to impact SPR kinetics, under fully dark-adapted conditions.

Regional differences in the rate of rise of the photon response in frog and toad could originate from an axial concentration gradient of transducin. A significant fraction of transducin exits the ROS for the inner segment following exposure to bright light, slowly returning only after a prolonged period in darkness (Sokolov et al., 2002). With as many as 2000 disks impeding axial diffusion within a toad ROS, transducin levels might not equilibrate fully even after dark adaptation overnight. Biochemical quantification of transducin showed that in dark adapted rats, for which ROS length is half that in toad, transducin levels at the tip of the ROS appear to be slightly lower than at the base (Sokolov et al., 2002). By reducing the rate of transducin activation by R*, a lower transducin concentration could account for the reduced photon response at the distal tip of the ROS and its slower rising phase.

The faster recovery of the dim flash response elicited at the base of the toad ROS compared to the tip in the absence of bicarbonate could also be attributed in part, to an increasingly higher concentration of bound or free Ca^2+^ along the ROS with distance from the base (Leibovic, 2001; Li et al., 2020). High Ca^2+^ would slow the recovery phase of the SPR by delaying the shutoff of photoexcited rhodopsin and by suppressing membrane guanylate cyclase activity (Gross and Wensel, 2011; Wen et al., 2014; Ingram et al., 2016). Higher Ca^2+^ at the tip could arise from preferential Ca^2+^ release from distal disks (Li et al., 2020). In addition, Na^+^ is removed from the rod by Na^+^K^+^-ATPases in the inner segment (Stahl and Baskin, 1984; Ueno et al., 1984), so the tip may have a slightly higher concentration of Na^+^ and be somewhat depolarized relative to the base. The Na^+^/Ca^2+^,K^+^ exchanger in the ROS is voltage-dependent and sensitive to the concentration of Na^+^ inside (Lagnado et al., 1988), so the tip will have a lowered rate of Ca^2+^ extrusion. We captured the SPR base vs. tip differences in toad rods with our model by simulating base and tip as separate rods. Rod-t, for the tip, had a lower rate of transducin activation and an increased fraction of dark current carried by Ca^2+^ by 56%, which raised Ca^2+^ in darkness at the tip by 60% (see Supplementary Table S2).

Unexpectedly, bicarbonate greatly diminished the base to tip differences (Figures 2C,D, 3, 5, 6) and thus reduced SPR variability. The gradient reduction in the rising phase happened because of greater acceleration of the dim flash response by bicarbonate at the tip of the ROS, rather than a slowing at the base. Bicarbonate can alkalinize the ROS by combining spontaneously with a proton and subsequently releasing CO_2_, which is membrane soluble and will diffuse away. Raising pH up to about 8.8 would have contributed to the increase in dark current with bicarbonate (Sampath and Baylor, 2002; Duda et al., 2015). Elevated pH would also have promoted Ca^2+^ extrusion by the Na^+^/Ca^2+^, K^+^ exchanger (Hodgkin and Nunn, 1987) to a greater extent at the base, but such mechanisms cannot readily account for faster flash response kinetics at the tip. Acceleration at the tip must act on the initial steps of phototransduction involving the sequential activation of transducin and phosphodiesterase and subsequent CNG channel closure. Since bicarbonate and low Ca^2+^ stimulate guanylate cyclase to increase cGMP levels in darkness, as evidenced by the enhanced saturating response amplitude (Figure 4) and PDE activity is greatly dependent on substrate availability (Granovsky and Artemyev, 2000), a faster rate of cGMP hydrolysis should steepen the rising phase of the photon response and shorten its effective time. We tested this hypothesis utilizing our space-resolved biophysical model. The model predicted faster rising phases of flash responses at the base and tip but a selective reduction in cascade activity at the base (or an increase at the tip) was required in order to reduce the base to tip differences (see Supplementary Table S2; Figure 7). It is not clear why bicarbonate failed to influence the SPR rising phase in salamander rods using full field flashes (Figures 1C–F; see also Duda et al., 2015; Makino et al., 2019). Perhaps the effect was simply too small for detection (*cf.*, Figures 1A,B).

For toad rods, Baylor et al. (1979b) reported a CV at the peak of the SPR of ~0.2 in the absence of bicarbonate. However, they restricted photic stimulation to a central segment of the ROS, excluding the SPR variability arising from differences at the base and tip. Thus, the true CV for the SPR across the entire ROS would be considerably greater. Yet, behavioral tests of toads snapping towards moving dummy worms or preferring to jump toward dim green light indicate that their brains can interpret single photon signaling (Aho et al., 1993; Yovanovich et al., 2017). Our results provide an explanation; bicarbonate in the living eye plays an essential role in making photon counting possible by reducing the axial variability. In future studies, it will be interesting to unravel the basis for the axial SPR differences in the absence of bicarbonate in toad rods, to explain how bicarbonate improves SPR reproducibility, to explore whether additional species differences unrelated to ROS structure contribute to the different responses to bicarbonate in toad and salamander, and to examine whether bicarbonate contributes to or reduces axial SPR variance in mammalian rods with 3-5-fold thinner ROS (Nickell et al., 2007; Gilliam et al., 2012) and different incisure structures (Cohen, 1965).

## Data availability statement

The raw data supporting the conclusions of this article will be made available by the authors, without undue reservation.

## Ethics statement

The animal study was reviewed and approved by the Animal Care and Use Committee, Boston University.

## Author contributions

CM and PG: conceptualization, performed electrophysiology recordings and wrote the original draft. CM: funding acquisition. GC: performed the simulations. PG, GC, and CK: performed formal analysis. All authors: revised and edited. All authors with the exception of ED have approved the final version of the manuscript and have agreed to be accountable for all aspects of the work, ensuring that questions related to the accuracy or integrity of any part of the work were appropriately investigated and resolved.

## Funding

This work was supported by the National Science Foundation, DMS 1812601 and the National Eye Institute, EY011500, EY023980, and EY031702. The authors are solely responsible for the contents of this publication, which do not necessarily represent the official views of the National Institutes of Health or those of the National Science Foundation.

## Conflict of interest

The authors declare that the research was conducted in the absence of any commercial or financial relationships that could be construed as a potential conflict of interest.

## Publisher’s note

All claims expressed in this article are solely those of the authors and do not necessarily represent those of their affiliated organizations, or those of the publisher, the editors and the reviewers. Any product that may be evaluated in this article, or claim that may be made by its manufacturer, is not guaranteed or endorsed by the publisher.

## Footnotes

^1^ https://www.socscistatistics.com/tests/signedranks/default.aspx

## Supplementary material

SUPPLEMENTARY FIGURE S1 Test for linearity of responses to flashes through the slit. **(A)** Responses of a salamander rod in 30 mM bicarbonate to dim flashes at the base of its ROS. **(B)** Responses of the rod in **A** to flashes at its ROS tip. **(C)** Responses from **A** and from **B**, normalized to their respective peak amplitudes. **(D)** Responses of a toad rod in Ringer’s to flashes at the ROS base. **(E)** Responses of the rod in **D** to flashes at the ROS tip. **(F)** Responses from **D** and from **E**, normalized to their respective peak amplitudes. For each panel, the two flash strengths at 500 nm varied by ~two-fold.
