# Supplementary material for: Effects of cell size and bicarbonate on single photon response variability in retinal rods
Source: Front Mol Neurosci. 2022 Dec 14;15:1050545. doi: 10.3389/fnmol.2022.1050545 (PMC9796569; doi:10.3389/fnmol.2022.1050545)
Supplement: Supplementary file 1 [file Table_1.pdf]

Table S1. Parameters for salamander ROS.

Definition	Units	Value in Ringer's	Value in Bicarbonate
Maximal rate of cGMP synthesis at low $[Ca^{2+}]$	$\mu M\ s^{-1}$	50	100
Ratio of cGMP synthesis*:* high to low $[Ca^{2+}]$	-	0.02	0.0107
Incisure area per disk	μm^2	0.8	0.8
Basal rate of cGMP hydrolysis by PDE in darkness	s^{-1}	7.53×10^{-5}	7.53×10^{-5}
Buffering power for cGMP in the cytoplasm	-	1	1
Buffering power for Ca^{2+} in the cytoplasm	-	20	20
[cGMP] in darkness	μM	1.14	1.23
$[Ca^{2+}]$ in darkness	nM	815	991
Diffusion coefficient of cGMP	$\mu m^2\ s^{-1}$	160	160
Diffusion coefficient of Ca^{2+}	$\mu m^2\ s^{-1}$	15	15
Diffusion coefficient of E*	$\mu m^2\ s^{-1}$	0.8	0.8
Diffusion coefficient of T*	$\mu m^2\ s^{-1}$	1.5	1.5
Diffusion coefficient of R*	$\mu m^2\ s^{-1}$	0.7	0.7
Disk thickness	nm	14	14
Volume-to-surface ratio	nm	7	7
Faraday's constant	$C\ mol^{-1}$	96500	96500
Fraction of cGMP-activated current carried by Ca^{2+}	-	0.17	0.17
Height of ROS	μm	22.4	22.4
Dark current	pA	76.34	86.24
Maximum CNG channel current	pA	7000	7000
Saturated exchanger current	pA	17	17
Surface rate of cGMP hydrolysis by dark-activated PDE	$\mu m^3\ s^{-1}$	7.53×10^{-5}	7.53×10^{-5}
Surface rate of cGMP hydrolysis by light-activated PDE	$\mu m^3\ s^{-1}$	0.5	0.5
Rate constant for PDE* inactivation	s^{-1}	0.30	0.30

Kinetic constant of T*-E binding and thus E*production	$\mu m^2\ s^{-1}$	1	1
Half-saturating [Ca^{2+}] for guanylate cyclase activity	nM	135	135
[cGMP] for half-maximal CNG channel opening	μM	20	20
[Ca^{2+}] for half-maximal exchanger rate	μM	1.5	1.5
Incisure width	nm	15	15
Incisure length	μm	4.6377	4.6377
Ratio of interdiskal space to disk thickness	-	1	1
Interdiskal space	nm	14	14
Rate of T* formation by R*	s^{-1}	185	185
Number of disks	-	800	800
Number of incisures	-	23	23
Avogadro number	# mol^{-1}	6.02×10^{23}	6.02×10^{23}
Hill coefficient for Ca^{2+} dependence of guanylate cyclase activity	-	3	3
Hill coefficient for CNG channels	-	1.6	1.6
Surface density of active PDE in darkness	# μm^{-2}	100	100
Rod radius	μm	5.5	5.5
Ratio of outer shell thickness to disk thickness	-	15/14	15/14
Distance separating disk rim from plasma membrane (outer shell thickness)	nm	15	15
Lateral surface area of ROS	μm^2	773.5	773.5
Cytoplasmic volume	μm^3	1076	1076
