# Supplementary material for: Effects of cell size and bicarbonate on single photon response variability in retinal rods
Source: Front Mol Neurosci. 2022 Dec 14;15:1050545. doi: 10.3389/fnmol.2022.1050545 (PMC9796569; doi:10.3389/fnmol.2022.1050545)
Supplement: Supplementary file 2 [file Table_2.pdf]

Table S2. Parameters for toad rods.

Definition	Units	Value in Ringer's	Value in Bicarbonate
Maximal rate of cGMP synthesis at low [Ca^{2+}], base	μM s^{-1}	50	100
Maximal rate of cGMP synthesis at low [Ca^{2+}], tip	μM s^{-1}	50	65
Ratio of α from high to low [Ca^{2+}], base	-	0.02	0.01
Ratio of α from high to low [Ca^{2+}], tip	-	0.02	0.0167
Incisure area per disk, base	μm^2	0.486	0.486
Incisure area per disk, tip		0.525	0.525
Basal rate of cGMP hydrolysis by PDE in darkness	s^{-1}	7.53×10^{-5}	7.53×10^{-5}
Buffering power for cGMP in cytoplasm	-	1	1
Buffering power for Ca^{2+} in cytoplasm	-	20	20
[cGMP] in darkness, base	μM	1.16	1.22
[cGMP] in darkness, tip	μM	1.04	1.12
[Ca^{2+}] in darkness, base	nM	900	1030
[Ca^{2+}] in darkness, tip	nM	1450	1080
Diffusion coefficient of cGMP	μm^2 s^{-1}	160	160
Diffusion coefficient of Ca^{2+}	μm^2 s^{-1}	15	15
Diffusion coefficient of E*	μm^2 s^{-1}	0.8	0.8
Diffusion coefficient of T*	μm^2 s^{-1}	1.5	1.5
Diffusion coefficient of R*	μm^2 s^{-1}	0.7	0.7
Disk thickness	nm	14	14
Volume-to-surface ratio	nm	760	760
Faraday's constant	C mol^{-1}	96500	96500
Fraction of cGMP-activated current carried by Ca^{2+}, base	-	0.3	0.3
Fraction of cGMP-activated current carried by Ca^{2+}, tip	-	0.47	0.35
Height of ROS	μm	60	60

Dark current, base	pA	48	52
Dark current, tip	pA	43	46.5
Maximum CNG channel current	pA	4000	4000
Saturated exchanger current	pA	17	17
Surface rate of cGMP hydrolysis by dark-activated PDE	$\mu m^3\ s^{-1}$	7×10^{-5}	7×10^{-5}
Surface rate of cGMP hydrolysis by light-activated PDE	$\mu m^3\ s^{-1}$	0.5	0.5
Rate constant for PDE* inactivation	s^{-1}	0.48	0.48
Kinetic constant of T*-E binding and thus E*production	$\mu m^2\ s^{-1}$	1	1
Half-saturating [Ca^{2+}] for GC activity	nM	135	135
[cGMP] for half-maximal CNG channel opening	µM	20	20
[Ca^{2+}] for half-maximal exchanger rate	µM	1.5	1.5
Incisure width	nm	21.6	21.6
Incisure length, base	µm	2.5	2.5
Incisure length, tip		2.7	2.7
Ratio of interdiskal space to disk thickness	-	1	1
Interdiskal space	nm	14	14
Maximal rate of T* formation by R*, base	s^{-1}	480	336
Maximal rate of T* formation by R*, tip	s^{-1}	250	250
Number of disks	-	2143	2143
Number of incisures	-	18	18
Avogadro number	# mol^{-1}	6.02×10^{23}	6.02×10^{23}
Hill coefficient for GC effect	-	3	3
Hill coefficient for CNG channels	-	1.6	1.6
Surface density of active PDE in darkness	# μm^{-2}	100	100
Disk radius	µm	3	3
Ratio of outer shell thickness to	-	15/14	15/14

disk thickness			
Distance separating disk rim from plasma membrane (outer shell thickness)	nm	10.8	10.8
Lateral surface area of a ROS	μm^2	1131	1131
Cytoplasmic volume	μm^3	860	860
