# Supplementary material for: Effects of cell size and bicarbonate on single photon response variability in retinal rods
Source: Front Mol Neurosci. 2022 Dec 14;15:1050545. doi: 10.3389/fnmol.2022.1050545 (PMC9796569; doi:10.3389/fnmol.2022.1050545)
Supplement: Supplementary file 3 [file Table_3.pdf]

Table S3. Kolmogorov-Smirnov significance test for goodness of fits in Fig. 6.

Fig	trial 1 p-value	trial 2 p-value	trial 3 p-value	mean p-value
**6A**	0.098	0.096	0.097	0.097
**6B**	0.562	0.562	0.565	0.563
**6C**	0.916	0.917	0.917	0.917
**6D**	0.339	0.340	0.338	0.339

Kolmogorov-Smirnov tests were performed using 100,000 Monte Carlo samples per trial. Trials were conducted independently, in triplicate. $P < 0.05$ was considered to indicate significant disagreement between the model prediction and the data.
