# Supplementary figures and images for: Effects of cell size and bicarbonate on single photon response variability in retinal rods
Source: Front Mol Neurosci. 2022 Dec 14;15:1050545. doi: 10.3389/fnmol.2022.1050545 (PMC9796569; doi:10.3389/fnmol.2022.1050545)

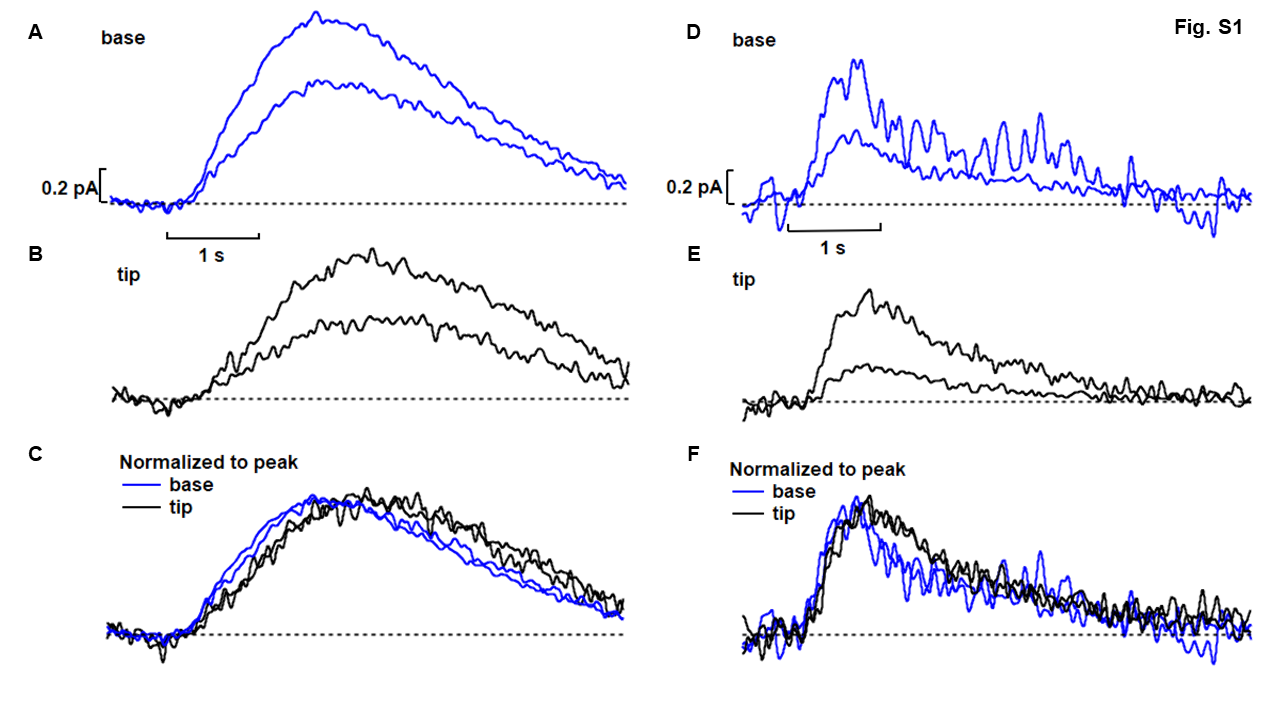

Supplement: SUPPLEMENTARY FIGURE S1 — Test for linearity of responses to flashes through the slit. (A) Responses of a salamander rod in 30 mM bicarbonate to dim flashes at the base of its ROS. (B) Responses of the rod in A to flashes at its ROS tip. (C) Responses from A and from B, normalized to their respective peak amplitudes. (D) Responses of a toad rod in Ringer’s to flashes at the ROS base. (E) Responses of the rod in D to flashes at the ROS tip. (F) Responses from D and from E, normalized to their respective peak amplitudes. For each panel, the two flash strengths at 500 nm varied by ~two-fold. [file Image_1.tif]
